# Study of Protective Layers Based on Crosslinked Glutaraldehyde/3-aminopropyltriethoxysilane

**DOI:** 10.3390/polym14040801

**Published:** 2022-02-18

**Authors:** Alessandro Pistone, Cristina Scolaro, Consuelo Celesti, Annamaria Visco

**Affiliations:** 1 Department of Engineering, University of Messina, Contrada Di Dio, I-98166 Messina, Italy; cristina.scolaro@unime.it (C.S.); consuelo.celesti@unime.it (C.C.); 2 Institute for Polymers, Composites and Biomaterials, CNR-IPCB, Via P. Gaifami 18, 9-95126 Catania, Italy

**Keywords:** coating, (3-aminopropyl)-triethoxysilane, glutaraldehyde, wet ability, adhesion, mechanical properties

## Abstract

In this paper, we report the synthesis and characterization of novel coatings based on (3-aminopropyl)-triethoxysilane (AP) mixed with different amounts of glutaraldehyde (GA). The synthesized coatings have been layered on a glass substrate and characterized by optical microscopy and roughness measurements, thermogravimetric analyses and differential scanning calorimetry, contact angle analysis, rheological measurement, and an adhesion test. It was observed that the higher the GA content (up to AP:GA ratio of 0.3), the sooner the crosslinking reaction starts, leading to a coating with increased hydrophobic and adhesion features without compromising the final AP cross-linked network. Hence, the obtained results show the effectiveness of AP modification with GA from the perspective of an application as protective coatings.

## 1. Introduction

Biofouling is one of the main worldwide problems associated with boats because of its accumulation on the hull surface [1,2]. Biological fouling is the main cause of coating deterioration, which ultimately leads to bio-corrosion, affecting roughness and strength, resulting in a huge increase in fuel consumption [3]. Most of the biocides used are potentially harmful to the environment due to the high rate of release into the aquatic environment [4,5]. In recent decades, much effort has been devoted to the development of efficient antifouling coatings and many studies have focused on advances in environmentally friendly antifouling coating techniques, using the principle of release of control biocides for marine applications [6,7,8,9].

Many types of siloxane compounds and formulations are currently widely investigated, such as poly-(dimethyl siloxane) (PDMS) [10,11,12,13,14,15,16,17,18]. However, the downside of poly (dimethyl siloxane) coatings has always been their poor adhesion and mechanical properties. This aspect results in easy damage, e.g., during normal ship handling and navigation, reducing performance and lifetime, with the formation of local unprotected areas containing a decrease of hydrophobic character and adsorption of proteins, polysaccharides, and glycoproteins that can easily lead to the solid attachment of barnacles [19]. To improve the adhesion and durability (i.e., mechanical properties) of siloxane-based coatings while maintaining their outstanding antifouling properties, many synthetic pathways have been explored, i.e., the application of primers to improve adhesion, the incorporation of additional inorganic and antifouling fillers, and the introduction of poly (urethane) and/or epoxy segments [20]. Verma et al. developed epoxy–polydimethylsiloxane based nanocomposite coatings with the inclusion of graphene oxide nanosheets, obtaining an improvement of hydrophobic character with water contact angle ranging from 90.1° to 115.2 and pull-off adhesion strength ranging from 1.32 to 5.94 MPa [21]. A nanocomposite coating based on polydimethylsiloxane and ZnO (PDMS-ZnO) was also prepared by Arukalam et al. The further addition of per-Fluoro-Decyl-Trichloro-Silane (FDTS) to PDMS-ZnO allows to obtain an antifouling coating with a water contact angle of 120° and pull-off adhesion strength of 2.41 MPa [22].

Another strategy to impart adhesion resistance is the functionalization of surfaces with 3-aminopropyltriethoxysilane [23]. Due to its additional amine functionality, 3-aminopropyltriethoxysilane is a particularly interesting bifunctional silane. In fact, 3-aminopropyltriethoxysilane is an organofunctional monosilane in which one side of the structure has an active group (amino and vinyl), which can react with synthetic resin molecules, such as epoxy, phenolic, or polyester. The other side of the structure has an alkoxy group that can react with the hydroxyl groups on the surface of the substrate and generate reactive silicon alcohol in the presence of water (aqueous solution) or air. So, this alkoxide can form a silicon oxide three-dimensional network in which there is the possibility of bonding through the Si–OH and the NH_2_ end groups. It can be used in the production of plastic reinforced with fiber in order to improve its mechanical strength, compressive strength, resistance to moist environments, as well as the wettability and dispersion of polymer fillers [24,25]. In addition, it is often used as a coupling agent for bonding organic molecules to hydroxylated silicon oxide or metal oxide substrates, due to the presence of the terminal amino group on the propyl chain, through reaction with glutaraldehyde, a bifunctional aldehyde. These aldehyde groups easily react with amine groups of the 3-aminopropyltriethoxysilane and of the biomolecules to form amine linkages [26,27,28]. To our knowledge, glutaraldehyde is widely used as a coupling agent to immobilize proteins or other biological molecules to substrates previously treated with 3-aminopropyltriethoxysilane. No literature data were found regarding the use of glutaraldehyde as an adjuvant in the 3-aminopropyltriethoxysilane cross-linking step thanks to its reaction with the amino functionalities of 3-aminopropyltriethoxysilane or about its effect on the chemical and mechanical properties of 3-aminopropyltriethoxysilane polymeric network. In this study, a three-dimensional network of 3-aminopropyltriethoxysilane was synthesized by using glutaraldehyde as an additional cross-linking agent thanks to its ability to bind easily to amino groups of 3-aminopropyltriethoxysilane. The synthesized coatings were characterized by optical microscopy, roughness measurements, thermogravimetric analyses and differential scanning calorimetry, contact angle analysis, rheological studies, and adhesion tests in order to investigate the effect of glutaraldehyde loading on the morphological and mechanical properties of the glutaraldehyde/3-aminopropyltriethoxysilane coating.

## 2. Materials and Methods

### 2.1. Materials

The reagents (3-aminopropyl)-triethoxysilane (98%) (AP) and glutaraldehyde solution (C_5_H_8_O_2,_ 25% in H_2_O) (GA) were purchased from Sigma Aldrich (St. Louis, MO, USA). All reagents and solvents were used without further purification.

Glutaraldehyde/3-aminopropyltriethoxysilane samples (code: AP/GA) with different AP:GA ratio were synthesized fixing the amount of AP to 1 mL (0.0045 mol) and varying the amount of GA to 0.3 mL (0.00075 mol), 0.1 mL (0.00025 mol), 0. 05 mL (0.00013 mol), 0. 03 mL (0.00008 mol), and 0.01 mL (0.0000275 mol). Solutions of AP and GA were maintained at room temperature under magnetic stirring for 30 min.

**Figure 1:**
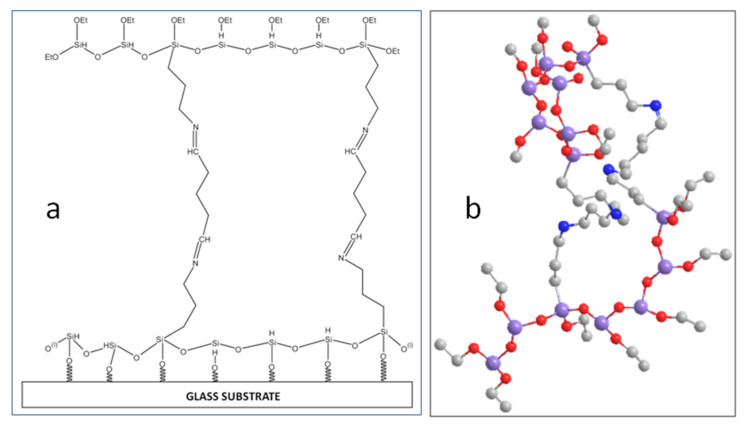
Chemical reaction of 3-aminopropyltriethoxysilane (AP) and Glutaraldehyde (GA) layered on a glass substrate (**a**); 3D view of AP/GA network; purple: silicon atoms (-Si-); reds: oxygen atoms (-O-); gray: carbon atoms (-C-); blue: nitrogen atoms (-N-) (**b**).

After that, the samples were applied on the glass supports, previously activated with a solution constituted of H_2_SO_4_/H_2_O_2_ in a 3:1 ratio and left to crosslink for the next 12 h, yielding in order the samples AP/GA 0.3, AP/GA 0.1, AP/GA 0.05, AP/GA 0.03, and AP/GA 0.01 (Figure 1a). The 3D cross-linked network of AP/GA is represented in Figure 1b.

### 2.2. Characterization and Testing

The morphology of the samples was investigated using a Hirox Digital Microscope KH-8700 (Hirox, Tokyo, Japan) optical microscope in mappings and xyz (3D) mode. Optical images were recorded using an MX (G) -5040Z lens at room temperature. The surface roughness (Ra) was calculated by the portable and compact roughness tester, Surftest SJ-210-Series 178 (Mitutoyo S.r.l., Milan, Italy):

(1) $$Ra=\frac{1}{N}\overset{n}{\underset{i=1}{\sum }}\left|Yi\right|$$

where *Ra* represents the arithmetic mean of the absolute values of the deviations of the evaluation profile (Yi) from the mean line. The measurement conditions were set according to the JIS2001 roughness standard, five sampling lengths, lengths of cut-off (λs = 2.5 μm, λc = 0.8 μm), and a stylus translation speed of 0.5 mm/s. Four roughness profiles per type of sample were performed and then an average profile was obtained.

Thermogravimetric analysis (TGA) was performed using the TAQ500 instrument (TA Instruments, New Castle, DE, USA) under argon flow at a flow rate of 100 mL/min. All samples tested, after a vacuum drying procedure, were heated up to 800 °C with a heating rate of 20 °C/min.

Differential scanning calorimetry (DSC) was performed using the TAQ500 instrument (TA Instruments, New Castle, DE, USA) under nitrogen flow at a flow rate of 50 mL/min, from room temperature to 550 °C, with a heating rate of 10 °C/min.

The wettability of the samples was evaluated by the contact angle “theta” (θ) measurement (according to the international standard ASTM D7334 [29]), (Prototype, Messina, Italy) by depositing a drop of deionized water (1 μL) on the horizontal surface of the sample, by means of a microlithic syringe (Hamilton, 10 μL). Contact angles θ have been evaluated by the sessile drop method (ASTM D7334) [30,31]:

(2) $$\theta_{w}=2\mathrm{arctg}\left(\frac{2h}{d}\right)$$

(3) $$\theta_{Y}=\mathrm{arcos}\;\left(\frac{{\cos\theta }_{w}}{r}\right)$$

where d is the diameter and h the height (both in mm) of the drop, θ_w_ is the Wenzel angle apparent dependent on the roughness of the surface, *r* is the surface roughness, and θ_Y_ is the Young contact angle of equilibrium on perfectly smooth surface. Measurements have been performed on AP/GA coatings deposited on DH36 steel metal substrate previously coated with a commercial primer, Jotacote Universal N10 two-component epoxy with polyamine hardener (3:1), and tiecoat Safeguard Universal ES, a two-component epoxy vinyl with a polyamide hardener (5:1) (supplied by Jotun Italia Srl). For each coating, the resulting contact angle is the average value of approximately 10–20 measurements made with deionized water.

Rheological property measurements were carried out by means of a rotational rheometer (Mod. SR5, Rheometric scientific, Piscataway, NJ, USA) with parallel plate geometry at room temperature. A dynamic stress sweep test (frequency of 1 Hz) was performed within the stress range of 0.5–1000 Pa to check the linear viscoelastic region (LVR), in which the storage elastic modulus, G’, and the viscous loss modulus, G’’, are independent of the applied shear stress. From this test, we choose the stress value of 4 Pa, within the LVR, to test our coatings, and according to the literature data [32]. Thus, frequency sweep tests (in stress control) were carried out in the frequency range 0.01–200 rad/sec at the constant stress value of 4 Pa. The rheological properties (frequency response of G’ and of G”) were checked 30 min after the cross-linking began. Each test was carried out in duplicate.

The adhesion test was performed using a Lloyd LR10K universal testing machine (Ametek-Lloyd Instruments Ltd., Fareham Hampshire, UK) in accordance with ASTM D4541-02 (or ISO4624:2016) by attaching a steel metal dolly perpendicularly on a DH36 steel metal sheet (80 mm × 10 mm, thickness 5 mm). The DH36 steel sheet had been previously fully coated with a first thin layer of commercial primer (Jotacote Universal N10, Jotun Italia Srl) (~143 μm thick) and with a second layer of tiecoat (Safeguard Universal ES, Jotun Italia Srl) (~323 μm thick). After the complete drying of the second tiecoat layer, a third layer of AP/GA coat, circular in shape (diameter ≈ 10 mm) was deposited as topcoat (~190 μm tick) under each metallic dolly. The dollies, before adhering to the topcoat, were rubbed, cleaned with a cotton swab dipped in alcohol, and then dried with a dry cloth. The pull-off test was performed with a LLOYD LR10K Universal Dynamometer machine (Load cell 10 KN, Preload 1.00 N, Speed 1 mm/min). Parameters analyzed were: *E* is the Young’s modulus (MPa); *σ_y_* is the yield stress; *ε_y_*% is the yield percentage deformation; *σ_max_* is the maximum stress (MPa); *σ_r_* is the stress at break (MPa); *ε_r_%* is the percentage deformation at break (%); *L_r_* is the load at break (N); *W_r_* is the work at break (J). Mechanical values are the result of the average calculated on 6 specimens for each type of top coat analyzed.

The mean differences and standard deviations of wettability, roughness, and adhesion test were calculated with Prism 8.4.1 (GraphPad Software, Inc., La Jolla, CA, USA). Data were first verified with the D’Agostino and Pearson test for the normality of the distribution and the Levene test for the homogeneity of variances. Data were normally distributed and homogenous. Therefore, they were statistically analyzed using one-way analysis of variance (ANOVA) and Bonferroni post hoc test for multiple comparisons at a level of significance set at *p* < 0.05.

## 3. Results and Discussion

**Figure 2:**
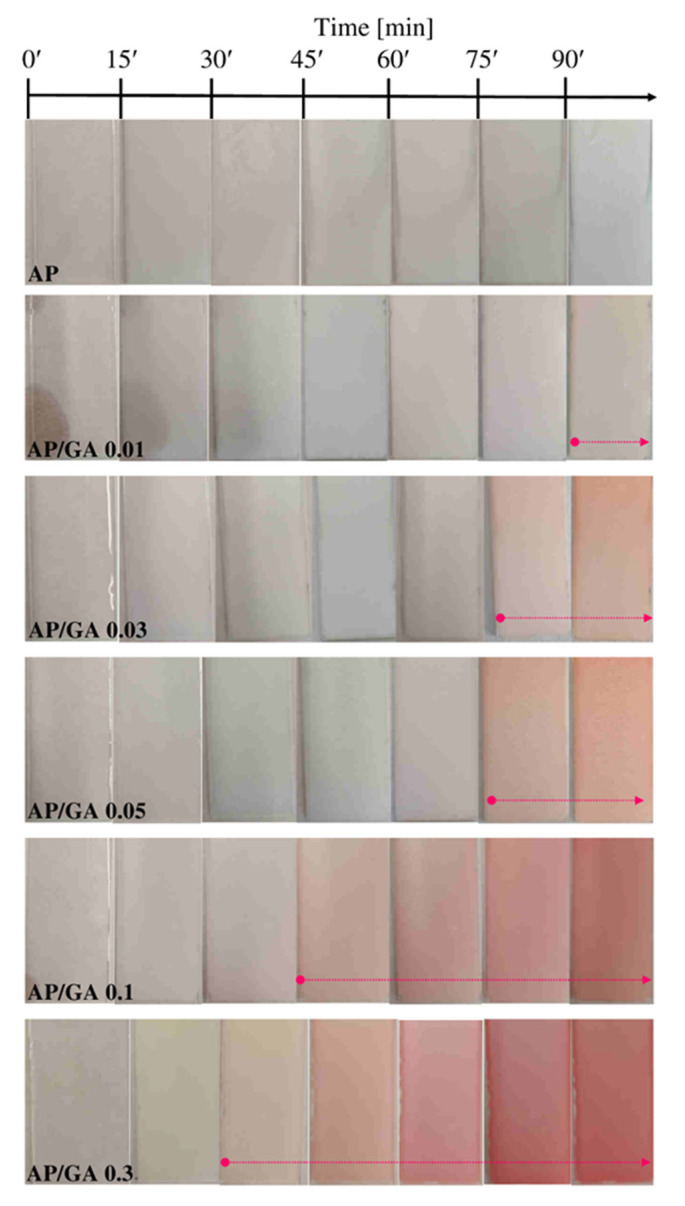
Photographs of the AP and the AP/GA coatings deposited on glass, taken every 15 min from the mixing of the two components.

In order to observe the chromatic change during the reaction between AP and GA in different amounts, the coatings were deposited on glass substrates as previously described in Figure 1a. The sequence of images of AP and AP/GA coating during their crosslinking reaction at room temperature is shown in Figure 2. Photographs were taken every 15 min from the mixing of the two components. The images of pure AP coating were considered as reference. All samples showed a uniform structure on the glass support, without cracking. The reaction between glutaraldehyde and 3-aminopropyltriethoxysilane gave rise to a change in color of the coating, from opaque white (typical of the AP coating) to pink, and hence to red in the hardened samples. The change in color appears much earlier as the glutaraldehyde content increases. In particular, the starting times are: ≥90 min in the AP/GA 0.01, ≥75 min in the AP/GA 0.03 and AP/GA 0.05, ≥45 min in the AP/GA 0.1, and about 30 min in the AP/GA 0.3 sample, as indicated by the arrows inside Figure 2.

**Figure 3:**
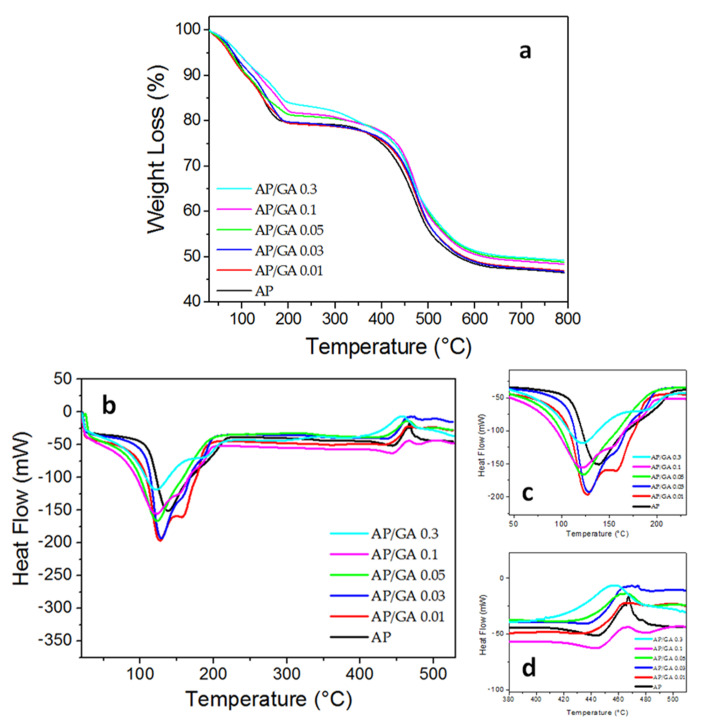
TG (**a**) thermograms and DSC (**b**–**d**) curves for AP, GA and AP/GA coatings. All experiments were performed under inert atmosphere.

The TGA-DSC analyses were carried out on AP/GA coatings at different amounts of GA and the thermal behavior was compared with the AP system. Thermal decomposition of the samples shows two main weight losses in the range of 50–200 °C, with a shoulder at 150 °C, and 350–600 °C (Figure 3a–d). The weight loss of analyzed samples in relation to the release of water molecules correlates with the temperature region below 200 °C. In this region, the weight loss decreases with the increase of GA content as well as the intensity of DSC peaks. The thermal degradation of the samples due to decomposition of the organic component corresponds to the higher temperature region. The weight loss and the DSC profiles between 350 and 600 °C show no significant difference by varying the GA content in the samples [33]. This result suggests that the reaction between aldehydic groups of bifunctional glutaraldehyde and amine groups of 3-aminopropyltriethoxysilane are not detrimental towards the final cross-linked structure of the samples.

**Figure 4:**
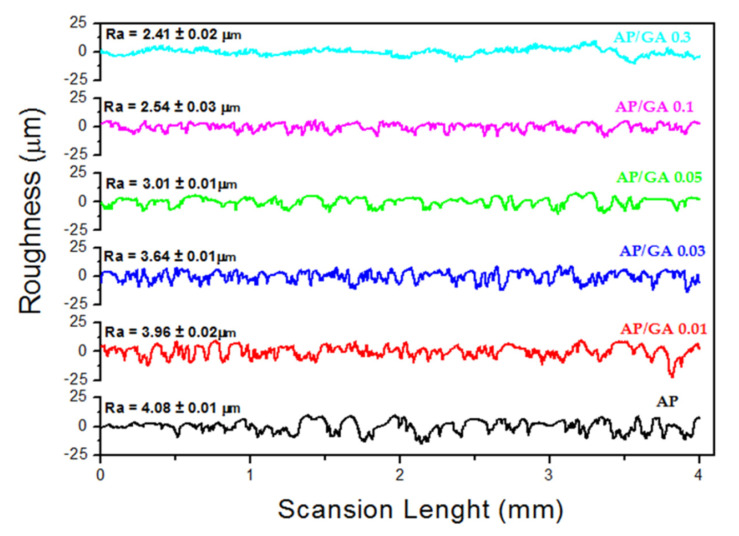
Surface roughness profiles of AP and AP/GA samples at different wt.% of GA.

The functionalization of AP chains with the aldehyde part leads to a highly significant decrease (*p* < 0.0001) in surface roughness compared to unmodified AP. Roughness (Ra) progressively decreases with the aldehyde concentration increasing (Figure 4) from 4.08 μm in the AP sample to 3.96 μm (*p* < 0.0001) in the AP/GA 0.01, to 2.41 μm (*p* < 0.0001) in the AP/GA 0.3 (see details of Ra in Figure 4).

**Figure 5:**
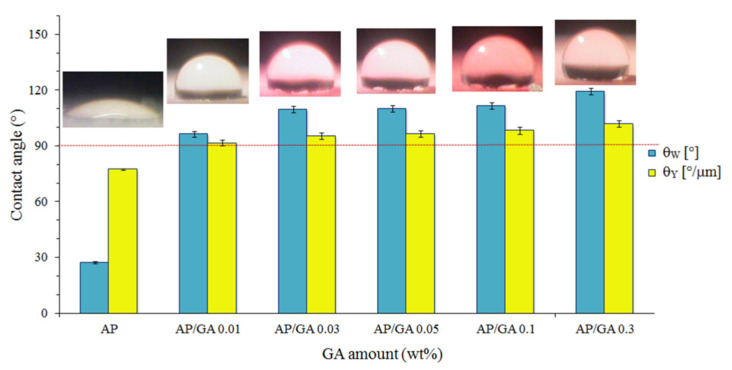
Comparison of contact angles θw and θ_Y_ of AP and AP/GA coatings at different GA content.

In the images associated with the graph of Figure 5 and Table 1, it is possible to see how all the AP/GA coatings exhibit contact angles (and therefore wettability) much higher compared to the AP coating whose contact angle is lower than 90°. The apparent static Wenzel contact angle θw depends on the morphology of the surface. Every surface, in fact, presents heterogeneities and when there is a full contact between the liquid and the substrate, or if there is air trapped inside the surface roughness, the measured contact angle of contact measured θw differs from the ideal one θ_Y_ (Young contact angle). Considering that hydrophilic coatings have a contact angle θ < 90° while the hydrophobic ones have θ > 90, the hydrophilic features of AP change after the treatment with glutaraldehyde in hydrophobic ones. Thus, both Young and Wenzel contact angles grow with the increasing GA amount with *p* < 0.0001. Furthermore, the comparison between the contact angles θw e θ_Y_ of the AP and AP/GA coatings at the different amount of GA shows a lower value of θ_Y_ with respect to θw for all coatings if normalized to roughness value, according to the Equation (3) (see Table 1).

**Figure 6:**
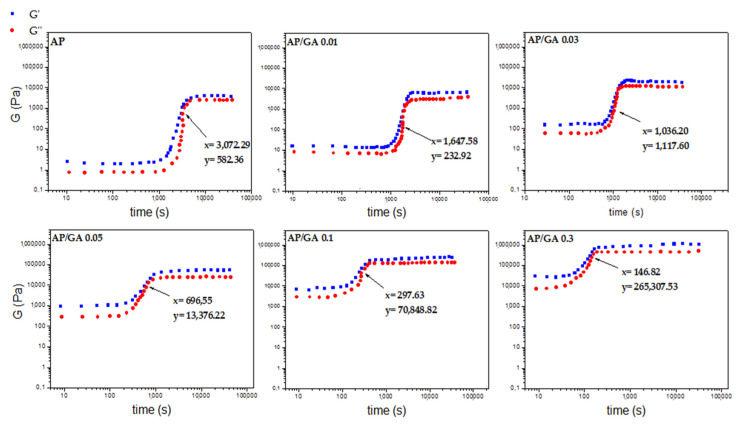
Cross-point between elastic and loss modulus (G’ and G”) vs. time of AP and AP/GA samples.

In Figure 6, the change in G’ and G’’ as a function of time is shown. Each gel point is highlighted by an arrow inside the graph itself. As known, the gel point represents the transition from the fluid state to the solid state. The gel point values are listed in Table 2. This phase change is due to the crosslink reactions taking place between AP and GA. Gel point increases from the value of 233 Pa (1648 s) in sample AP/GA 0.01 to the value of 70,849 Pascal reached in 298 s in the sample AP/GA 0.1. Therefore, an increase of an order of magnitude in the glutaraldehyde content (from AP/GA 0.01 to AP/GA 0.1) causes both an increase in the stiffness of the material and an increase in the crosslinking speed. Another further increase in glutaraldehyde content (from AP/GA 0.1 to AP/GA 0.3) increases the stiffness of the material, further shortening the crosslink reaction time (265,308 Pa and 147 s). Further addition of GA leads to an excessive embrittlement of the coating with the formation of cracks and detachments from the glass surface.

**Figure 7:**
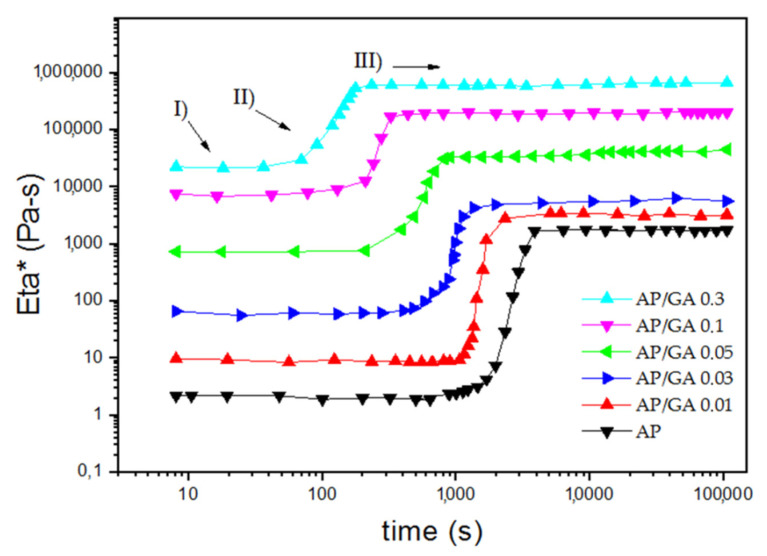
Complex viscosity (η*) vs. time of AP and AP/GA samples.

The quick increase in consistency of the polymer (due to the progressive reactions between the GA molecules with the AP which passes from the fluid state, to the gel state and finally to the solid state) can also be observed in the graph in Figure 7 (complex viscosity vs. time). Each curve can be distinguished into three parts: an initial plateau (zone I) in which the material has the lowest viscosity and still liquid consistency, a second transition phase (zone II) in which there is a sudden and rapid increase in viscosity, up to a further plateau (zone III) in which the material reaches the maximum hardening due to solidification after the completion of the cross link reaction. From the trend of the various curves, it can be easily seen that there is a marked increase in complex viscosity and a reduction in the gap between zone I and zone III as the GA content increases. This result is in agreement with the photographic images of Figure 2 which show the higher glutaraldehyde content leading to a progressive increase in the speed and times of crosslinking. Table 2 also indicates the value of the storage module G’ after 10,000 s of reaction between AP and GA. It grows from 3800 Pascal in the sample AP to 6300 Pascal in the sample AP/GA 0.01, almost doubling its initial value. Moreover, it increases again by an order of magnitude in the AP/GA 0.03 and AP/GA 0.05 samples (i.e., 20,569 Pa and 51,776 Pa, respectively), by two orders of magnitude in AP/GA 0.1 (236,828 Pa), and finally by three orders of magnitude in AP/GA 0.3 sample (about 1 MPa).

**Figure 8:**
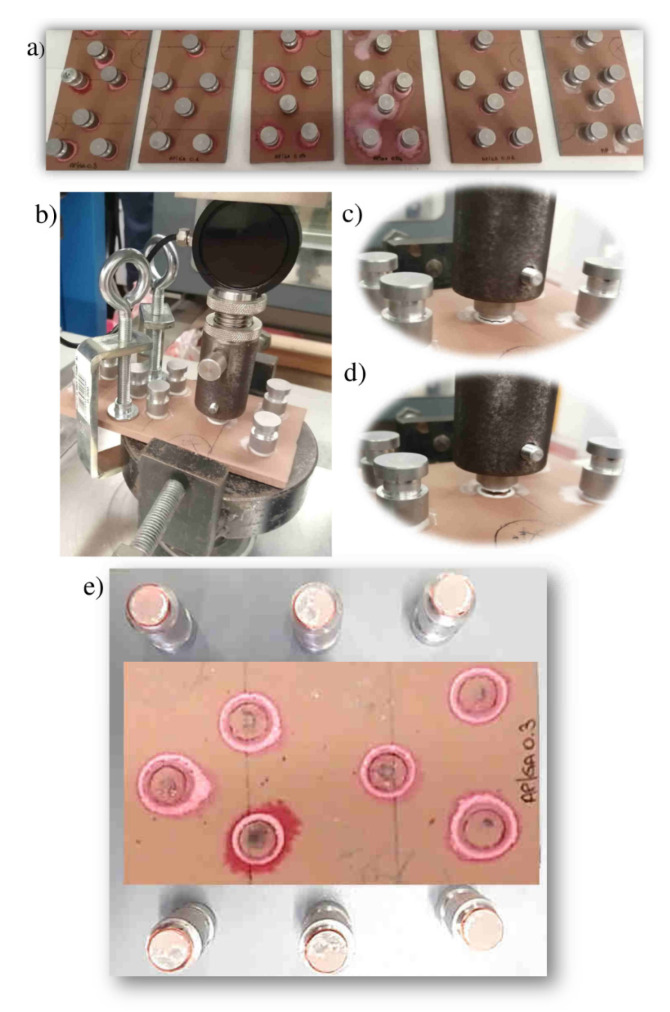
Pull-off Test: Image of the samples (**a**); fixing and stretching phase of the dolly in the dynamometer (**b**); step of breaking the specimen from the metal substrate (**c**,**d**); image of the metal specimen and the dolly after the pull off test for the sample AP/GA 0.3 (**e**).

The pull-off method was used to evaluate the mechanical adhesion resistance of the silica based coatings with the DH36 steel sheet. As detailed in Section 2, the test is performed by fixing small dollies perpendicular to the metallic specimens (Figure 8a).

After the paint has dried, a coupling connector from the dynanometer is attached to the dolly, which applies a tension perpendicular to the test surface (Figure 8b). The applied force is increased gradually and monitored until it becomes larger than the bond strength between the coating and the substrate: a fracture occurs and the dynamometer detaches the coating from the substrate (Figure 8c,d).

The nature and the type of fracture of the dolly from the test plan are carefully evaluated through visual inspection, according to ASTM D4541-02 or ISO4624:2016.

Figure 8e shows both the metal and dolly detached surface after the pull off test performed with the AP/GA 0.3 coating. The AP/GA 0.3 coating breaks at an average tensile stress of 1.7 MPa. Visual inspection of the fracture surface on both sides (dolly/substrate) reveals an adhesive type fracture occurring at the interface between the dolly and the liner. This type of adhesive fracture was also detected for all other sample series.

**Figure 9:**
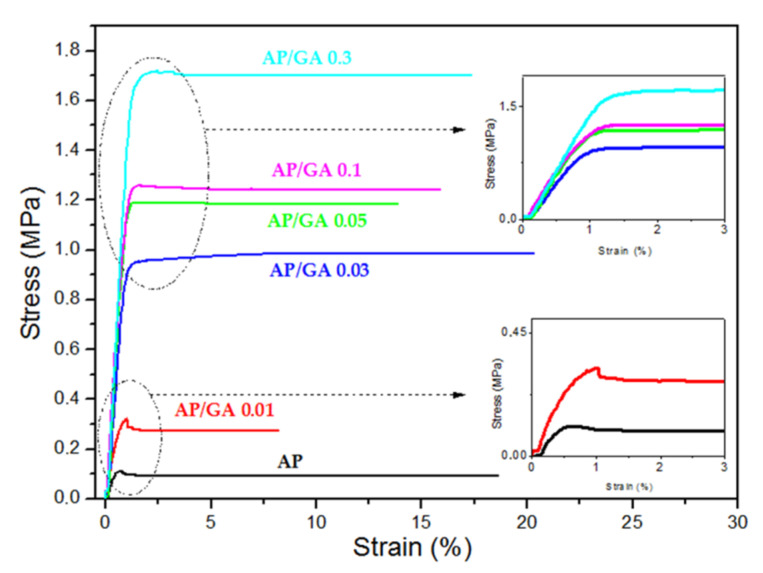
Stress/strain curves of AP and all the AP/GA coatings with a magnification of the yielding area in the inset graphs.

In the graph of Figure 9, the stress–strain curves of all the studied coatings are plotted. Mechanical parameters are given in detail in Table 3.

The adhesive behaviour of the AP and AP/GA 0.01 coating appears to be very close since both exhibit a yielding typical of ductile material at 0.11–0.32 MPa and at 0.72–1.03% of yielding stress and yielding strain, respectively. The yielding disappears in the coatings with higher GA contents (≥AP/GA 0.05), as can be observed in the inset magnified graphs.

The mechanical behaviour, highly significant (*p* < 0.0001), became that of a stiffer, stronger, and more tenacious coating compared to pure AP. We can notice as:

- Young modulus improves from 36–64 MPa, in the AP and AP/GA 0.01 coating (*p* = 0.3391, not significant), respectively, up to 167 MPa in the AP/GA 0.3 one (*p* < 0.0001);
- strength at break improves from 0.09–0.27 MPa, in the AP and AP/GA 0.01 coating, respectively (*p* < 0.0001), up to 1.7 MPa in the AP/GA 0.3 one (*p* < 0.0001);
- work at break improves from 0.013–0.016 J, in the AP and AP/GA 0.01 coating (*p* < 0.0001), respectively, up to 0.21 J in the AP/GA 0.3 one (*p* < 0.0001).

The adhesion test results evidenced that the presence of glutaraldehyde in the coatings induces a significant change in their mechanical resistance, due to the structural change confirmed by rheological data. The GA presence at the highest load (in AP/GA 0.3), indeed, increases the tensile strength and the elastic modulus of pure AP of 1788% and 363%, respectively (see details in Table 3). Therefore, in accordance with the rheological data, the presence of GA also has a fundamental role in the adhesive applications of the coating as it facilitates the crosslinking of AP.

## 4. Conclusions

In this study, (3-Aminopropyl)-triethoxysilane (AP) based coatings were modified by the addition of glutaraldehyde (GA), whose bifunctional aldehydic groups can easily bond to the amine group of 3-aminopropyltriethoxysilane, performing additional cross-linking reactions and leading to a different three-dimensional network than the 3-aminopropyltriethoxysilane alone.

The crosslinking reaction occurs first as the glutaraldehyde content increases. This can be observed both from the chromatic variations during the reaction which changes from opaque white, to pink, and then to red, as the degree of crosslinking increases. Alternatively, it can be observed from the gel point variation through rheological analyses which resulted in agreement with the chromatic variations.

The addition of GA up to an AP:GA ratio of 0.3 inside the 3-aminopropyltriethoxysilane structure does not compromise the intrinsic thermal properties of cross linked samples and leads to the best performing coating in terms of adhesion and wettability. In particular, the coating with the lowest GA amont (AP/GA 0.01) exibited a 0.27 MPa of tensile strength and a work at rupture of 20 N, while the highest one (AP/GA 0.3) showed up to 1.7 MPa of tensile strength and a work at rupture of 103 N (one order more). Higher GA ratios cause a degradation of the coating with the formation of cracks and detachments from the glass surface.

All the physical and mechanical characterization data have been verifed by statistical analysis and resulted significative.

Works are in progress to optimize the synthetic parameters for the preparation of coatings with AP:GA ratio higher than 0.3 and to investigate both physical and mechanical properties. Further biological assays will be pursued in order to evaluate the resistance against marine fouling.

## Figures and Tables

**Table 1:** Wenzel and Young contact angles of AP and AP/GA coatings.

Sample Code	θw (°)	θ_Y_ (°/μm)
AP	27.34 ± 0.90	77.41 ± 0.90
AP/GA 0.01	96.23 ± 3.02	91.57 ± 3.02
AP/GA 0.03	109.70 ± 3.35	95.37 ± 3.35
AP/GA 0.05	110.11 ± 3.60	96.55 ± 3.60
AP/GA 0.1	111.53 ± 3.70	98.31 ± 3.70
AP/GA 0.3	119.34 ± 3.49	101.75 ± 3.49

**Table 2:** Rheological parameters of the AP and AP/GA coatings.

Sample Code	Gel Point (Pa)	Time Gel Point (s)	G’ at 10,000 s (Pa)
AP	582	3072	3821
AP/GA 0.01	233	1648	6229
AP/GA 0.03	1118	1036	20,570
AP/GA 0.05	13,376	697	51,776
AP/GA 0.1	70,849	298	236,828
AP/GA 0.3	265,308	147	1,143,717

**Table 3:** Mechanical characterization of AP and AP/GA coatings.

Sample Code	E [MPa]	σ_y_ [MPa]	ε_y_ [%]	σ_r_ [MPa]	ε_r_ [%]	L_r_ [N]	W_r_ [J]
AP	36.11 ± 2.61	0.11 ± 0.01	0.71 ± 0.03	0.09 ± 0.005	18.61 ± 1.50	7.07 ± 0.67	0.0128 ± 0.0001
AP/GA0.01	64.70 ± 3.80	0.32 ± 0.02	1.03 ± 0.06	0.27 ± 0.02	8.21 ± 0.48	20.93 ± 1.66	0.0165 ± 0.0001
AP/GA0.03	120.19 ± 9.28	-	-	0.99 ± 0.07	20.32 ± 2.06	75.52 ± 7.26	0.1467 ± 0.0002
AP/GA0.05	155.44 ± 11.40	-	-	1.18 ± 0.08	13.87 ± 1.03	90.75 ± 5.20	0.1250 ± 0.0001
AP/GA0.1	163.68 ± 13.33	-	-	1.24 ± 0.47	15.88 ± 0.28	95.30 ± 10.35	0.1448 ± 0.0001
AP/GA0.3	167.48 ± 11.73	-	-	1.70 ± 0.51	17.38 ± 1.09	130.33 ± 7.07	0.2154 ± 0.0001

## Data Availability

Full data are available upon request to the corresponding author.
